# Do household perceptions influence enrolment decisions into community-based health insurance schemes in Tanzania?

**DOI:** 10.1186/s12913-021-06167-z

**Published:** 2021-02-19

**Authors:** Alphoncina Kagaigai, Amani Anaeli, Amani Thomas Mori, Sverre Grepperud

**Affiliations:** 1 Department of Health Management and Health Economics, University of Oslo, P.O. Box 0315, Oslo, Norway; 2 Department of Development Studies, Muhimbili University of Health and Allied Sciences, P.O. Box 65001, Dar es Salaam, Tanzania; 3 Department of Global Public Health and Primary Care, University of Berge, P.O. Box 7804, 5020 Bergen, Norway

**Keywords:** Community-based health insurance scheme, Cross-sectional household survey, Principal component analysis, Factor analysis, Perceptions factors

## Abstract

**Background:**

Several countries including Tanzania, have established voluntary non-profit insurance schemes, commonly known as community-based health insurance schemes (CBHIs), that typically target rural populations and the informal sector. This paper considers the importance of household perceptions towards CBHIs in Tanzania and their role in explaining the enrolment decision of households.

**Methods:**

This was a cross-sectional household survey that involved 722 households located in Bahi and Chamwino districts in the Dodoma region. A three-stage sampling procedure was used, and the data were analyzed using both factor analysis (FA) and principal component analysis (PCA). Statistical tests such as Bartlett’s test of sphericity, Kaiser-Meyer-Olkin (KMO) for sampling adequacy, and Cronbach’s alpha test for internal consistency and scale reliability were performed to examine the suitability of the data for PCA and FA. Finally, multivariate logistic regressions were run to determine the associations between the identified factors and the insurance enrolment status.

**Results:**

The PCA identified seven perception factors while FA identified four factors. The quality of healthcare services, preferences (social beliefs), and accessibility to insurance scheme administration (convenience) were the most important factors identified by the two methods. Multivariate logistic regressions showed that the factors identified from the two methods differed somewhat in importance when considered as independent predictors of the enrollment status. The most important perception factors in terms of strength of association (odds ratio) and statistical significance were accessibility to insurance scheme administration (convenience), preferences (beliefs), and the quality of health care services. However, age and income were the only socio-demographic characteristics that were statistically significant.

**Conclusion:**

Household perceptions were found to influence households’ decisions to enroll in CBHIs. Policymakers should recognize and consider these perceptions when designing policies and programs that aim to increase the enrolment into CBHIs.

**Supplementary Information:**

The online version contains supplementary material available at 10.1186/s12913-021-06167-z.

## Background

According to the World Health Organization (WHO), at least half the world’s population living in low- and middle-income countries (LMICs) lack access to essential health services [1]. Out-of-pocket health expenditures in these countries contribute to more than 40% of the total health budget [2] and more than 800 million people spend more than 10% of their household budget on healthcare [3]. Policymakers in LMICs are looking for strategies to improve access to health services, and the most important one has been the establishment of voluntary non-profit insurance schemes commonly known as Community Based Health Insurance Scheme (CBHIs), targeting rural populations and the informal sector [3, 4]. Such schemes are given different names such as; community health insurance [5, 6], micro health insurance [7, 8], community health funds (CHF) [9, 10], and mutual health organizations [11]. In Tanzania, the scheme is named the Community Health Fund (CHF) and in this paper, we will apply this concept.

In 1996, Tanzania piloted a “Community Health Fund” denoted as CHF, which was later scaled-up countrywide after showing promising results. CHF is a voluntary prepayment scheme that primarily provides access to primary care services. Before 2016, each district had different arrangements for the premium amount paid by each household per annum [12]. A total of 6–8 family members were covered under CHF and could receive the primary health services up to the district level from public health facilities only. The main rationale behind the establishment of CHF was the need to provide risk protection to rural residents and people working in the informal sector comprising more than 70% of the total national population [13]. Despite concerted efforts to promote the scheme, the enrolment rate has remained below expectations [14]. Various explanations for the low enrolment include unaffordable premiums, poor quality of services, poor scheme management, and lack of trust [9, 15].

In 2011, the Tanzanian government decided to reform the CHF and introduced an “improved Community Health Fund” (iCHF) as a pilot in the Dodoma region, with a flat annual premium of about 15 USD [8]. The iCHF included additional services such as x-rays, ultrasounds, and in-patient services (including major surgery) from both hospital levels (District and Regional). iCHF also simplified the enrolment process by using a mobile application (an insurance management information system). Services such as CT-scan, HIV services, screening for cancer, and other non-communicable diseases are exempted from the scheme. By 2018, the scheme was fully implemented in Dodoma and seven more regions. The government target was for at least 70% of the population to be covered by National Health Insurance Fund (NHIF) and iCHF by 2020 [16], which are the two main public insurance schemes. The future iCHF enrolment growth rate remains highly uncertain due to limited knowledge about its’ attractiveness to the informal sector. There is an urgent need to explore the factors that determine the enrolment behaviors of rural residents. Such information will enable policymakers to adjust implementation strategies before the scheme is rolled out at the national level.

Furthermore, this study tackles an important and relevant issue in the health insurance literature which is to understand the factors that influence CBHI in developing country’s context. This aspect is important for the achievement of the Sustainable Development Goal (SDG) 3, target 3.8: on the universal health coverage which includes; financial risk protection, access to quality essential healthcare services and access to safe, effective, quality and affordable essential medicines and vaccines for all.

Two previous studies have applied factor analysis when studying the role of household perceptions in association with insurance schemes in LMICs [17, 18]. The first one studied mixed urban-rural populations in Ghana and found scheme factors (price, benefits, and convenience) to be the most important ones [17]. The second study studied urban populations in India and identified “lack of awareness about the need for insurance” and “low and irregular income” as the most important barriers to enrolment [18]. Our study utilized an approach similar to the one used in Ghana and India when analyzing the role of perceptions towards the iCHF scheme in rural Tanzania. We apply both principal component analysis (*PCA*) and factor analysis (*FA*).^1^ The importance of the perception factors is determined by the amount of variation explained by them. To study the associations between the identified perception factors and the enrolment decision, multivariate logistic regressions will be used. In the following sections, we present the method used, followed by the presentation of the results and the concluding discussion.

## Methods

We used an observational cross-sectional study design to conduct a household survey in Bahi and Chamwino districts of Dodoma region in central Tanzania. This design was used because it provides a snap-shot evaluation of variables under investigation at a particular point in time.

### Study setting and sampling

Dodoma region consists of seven districts with a population of more than 2 million people according to the 2012 national population census where 10% live in Bahi and 15% in Chamwino [13]. The prime economic activity in both districts is agriculture and livestock keeping. Administratively, each district in Dodoma is divided into divisions, wards, and villages. Bahi is organized into 4 divisions, 22 wards, and 59 villages while Chamwino is divided into 5 divisions, 36 wards, and 107 villages. Furthermore, Bahi contains 6 primary health care centers and 37 primary care clinics (dispensaries) while Chamwino contains 1 hospital, 5 primary care centers, and 66 primary care clinics (dispensaries).

We used a multistage sampling technique to select wards and villages in each district. First, we used a purposive sampling technique to select 2 districts from Dodoma region. Then we selected wards from each division in the two districts. A total of 8 wards were selected from Bahi and 10 wards from Chamwino. Thereafter we selected two villages from each ward based on criteria such as health facility availability and location (16 villages from Bahi and 20 from Chamwino). At stage three, we employed systematic random sampling techniques in the selection of households.^2^ The first household was selected randomly from within the sampling frame. The office of the Executive Officer in each village was selected as a central point where the trained research assistants met. Each of the four trained interviewers walked in different directions (north, east, south, and west) and every third household was approached. The aim of doing this was to make sure that the population is evenly sampled and to obtain a good representative of the targeted population. The total sample size was 722 households (303 for Bahi and 419 for Chamwino). Data were collected from June to August in 2019 using a pre-tested structured questionnaire.

### Variables

Insurance status was our outcome variable with two categories; member (yes) and non-member (no) of iCHF. The possession of health insurance (iCHF) was determined by asking if the respondents were currently members of iCHF or not members. The explanatory variables in this study were the perception factors that influence an individual decision to enroll or not into the improved community health fund. The questionnaire (attached as Additional file 1) contained 38 questions/statements on household perceptions which were then subjected to factor analysis and principal component analysis to obtain factors to use as variables. The questions were formulated as statements and the respondents were asked to express their opinions by using a five-point Likert scale ranging from 1 (strongly disagree) to 5 (strongly agree). The choice of statements was inspired by the ones applied by Jehu-Appiah, and Kansra [17, 18] but also from previous literature that has been conducted on health insurance such as [5, 21, 22]. Of the 38 perception questions/statements, we included those for which we had a prior belief about the direction of the effects on the membership decision, thus leaving us with a total of 33 statements. These statements were then divided into three different groups (i) provider-related, (ii) preferences (beliefs and attitudes), and, (iii) scheme-related. The scheme-related statements were further subdivided into the following subgroups; convenience (access), recommendation, affordability, and understanding (information). Another category of explanatory variables was socio-economic variables and demographic characteristics. These variables were selected based on factors cited from different literature as factors that influence the individual decisions to enroll in health insurance such as [23, 24].

### Data analysis

A descriptive statistics summary was conducted on the socio-demographic household characteristics followed by factor analysis (FA) and principal component analysis (PCA) for the statements intended to measure household perceptions. The two methods were independently employed to demonstrate the robustness of our findings since the underlying assumptions differ. PCA assumes that there is no unique variance, the total variance is equal to common variance while FA assumes that total variance can be partitioned into common and unique variance [25].

Before performing PCA and FA, we conducted reliability, validity, and consistency tests. First, the Bartlett test of sphericity was calculated to test for correlations among the variables which showed that there was a correlation among variables. Second, the Kaiser-Meyer-Olkin measure (KMO), a test for sampling adequacy, was performed and found that the value of KMO > 0.5. Third, Cronbach’s Alpha test was performed to measure internal consistency and scale reliability which was > 0.7. Finally, logistic regressions were done to determine possible associations between the extracted factors and the membership status to iCHF. We chose to use the Logistic regression method because our outcome variable is a binary outcome (“Yes” for members and “No” for non-members). Data cleaning, validation, and all statistical analysis were performed using STATA 14.0 software.

## Results

The results are presented in three different subsections where the first presents the study population (descriptive statistics), the second present the results from PCA and FA methods, while the third presents the findings of the regression analyses.

### Characteristics of the study population

Tables 1 and 2 present some of the background characteristics of our respondents. Table 1 presents the mean and standard deviations of the background variables, while Table 2 shows how our outcome variable (membership status) differs across different explanatory variables. Our study consisted of 722 respondents, 304 (42.1%) of them being men while 418 (57.9%) were female. The mean age of the respondents was 44.7 years (SD. 13.67). Most of the respondents i.e. 72% had completed primary school education and almost three-quarter were engaged in small-scale farming. The mean household size was 5.4 members (SD. 2.3). Thirty-seven percent of the respondents had a monthly income below 50,000 Tanzanian shillings (TZS), which is equivalent to 22 USD, while 1% had a monthly income above 1 million TZS (435 USD). It also follows from Table 2 that 30% of the respondents reported that their households were enrolled in the iCHF as members, of which 61.5% were female and 39% were men.

**Table 1:** Characteristics of the study participants

Variables	Mean	SD
Age (years)	44.67	13.67
Household size	5.39	2.28
Monthly income (in TZS)	124,358	188,538
Sex (1 = female)	0.42	0.49
Marital status (1 = married)	2.67	1.37
Religion (1 = Christian)	0.86	0.35
Occupation (1 = farmer)	0.74	0.44
Education level		
No formal Education(1 = yes)	0.18	0.38
Primary Education (1 = yes)	0.72	0.45

Source: Authors’ calculation based on primary data

Note: Primary data were collected from two rural districts of Dodoma region (Bahi and Chamwino)

**Table 2:** Characteristics of the respondents by membership status

Characteristics	Member(s)(%)	Non-Member(s)(%)	Total
*Age (years)*			
60+	39 (17.9)	61 (12.10)	100 (13.9)
40–59	103 (47.2)	238 (47.2)	341 (47.2)
26–39	63 (28.9)	176 (34.9)	239 (33.1)
18–25	13 (5.9)	29 (5.8)	42 (5.8)
*Sex*			
Female	134 (61.5)	284 (56.4)	418 (57.9)
Male	84 (38.5)	220 (43.7)	304 (42.1)
*Education*			
Secondary and higher education	28 (12.8)	47 (9.3)	75 (10.4)
Primary education	154 (70.6)	366 (72.6)	520 (72)
No education	36 (16.5)	91 (18.1)	127 (17.6)
*Marital status*			
Unmarried	55 (25.2)	143 (28.4)	198 (27.4)
Married	163 (74.8)	361 (71.6)	524 (72.6)
*Household size*			
≥ 10	10 (4.6)	20 (4.0))	30 (4.2)
7–9	56 (25.7)	122 (24.2)	178 (24.7)
4–6	112 (51.4)	261 (51.8)	373 (51.7)
≤ 3	40 (18.4)	101 (20.0)	141 (19.5)
*Occupation*			
Non-farmer	58 (26.6)	129 (25.6)	173 (25.9)
Farmer	160 (73.4)	375 (74.4)	535 (74.1)

Source: Authors’ calculation based on primary data

Note: Primary data were collected from two rural districts of Dodoma region (Bahi and Chamwino)

### Principal component and factor analysis

We start by reporting the various statistical tests performed before PCA and FA. Results for Bartlett’s test of sphericity, Kaiser-Meyer-Olkin measure (KMO), and Cronbach’s alpha are reported in Table 3. According to the literature [26, 27], such diagnostic procedures indicate to what extent PCA and FA are appropriate. We observed that the standard requirements for KMO and Cronbachs alpha (see the right column of Table 3) were fulfilled.

**Table 3:** KMO measure, Cronbach’s alpha and Bartlett’s test of sphericity

S/N	Test	Values	Requirements
1	Kaiser-Meyer-Olkin (KMO) measure of sampling adequacy	0.815	KMO > 0.5
2	Cronbach’s alpha measure of scale reliability	0.801	α > 0.7
3	Bartlett’s test of sphericity		
	Chi-square	4892.747	
	Degrees of freedom	703	
	Significance	*p* < 0.000	*p* < 0.05

Source: Author’s illustration

Both *PCA* and *FA* apply eigenvalues higher than one as the inclusion criteria [28]. According to Costello and Osborne, variables whose loadings are ≥ |0.3| should be retained [25], We also carried out Orthogonal rotation (varimax) to improve the interpretation of the extracted factors.

Our findings on *PCA* are presented in Table 4. For this method, 10 factors met the eigenvalue criteria and they accounted for 60% of the explained variation. Three of the 10 factors did not fulfill the factor-loading criteria (two or more statements within each factor and a factor loading ≥ |0.3|), leaving us with seven factors that in sum contained 28 of the 33 statements. The number of statements belonging to each factor varied from two to six. The seven factors are quite homogenous in the sense that they include statements that are concerned with similar subjects. The exception is the two statements that are concerned with affordability (price-income considerations) that are grouped into Preferences (S*11*) and Knowledge (S*24*). We also observe that the 9 statements that measure the degree of understanding are grouped into three different factors denoted as Understanding, Knowledge, and Awareness.^3^ It follows that the most important factor is provider-related (Quality) since accounting for almost 11% of the explained variance. This factor includes statements that all measure various quality dimensions of health care services. The least important factors are the five scheme-related factors of which Convenience is the most important one (7% of the explained variance). Preferences are the second most important factor since explaining more than 9% of the variance. This factor reflects general preferences as well as alternative strategies to insurance (borrowing and saving) and curing (traditional medicine).

**Table 4:** Principal Component Analysis (*PCA*): Household perceptions towards iCHF

S/N	Factors and statements	The explained variance (%)	Factor Loadings
*P1*	*Quality (health care services)*	10.6	
S1	Healthcare services		0.76
S2	Healthcare personnel		0.72
S3	Long waiting time		−0.71
S4	Reasonable treatment time		0.71
S5	Discrimination of members		−0.65
S6	Availability of drugs		0. 56
*P2*	*Preferences (beliefs and priorities)*	9.5	
S7	iCHF is a loss of money		0.69
S8	I save money in case of illness		0.68
S9	I borrow money in case of illness		0.66
S10	Prefer traditional healers		0.59
S11	Low benefit-premium ratio		0.50
S12	Insurance brings bad luck		0.42
*P3*	*Convenience (iCHF accessibility)*	7.2	
S13	Office hours		0.83
S14	Opening location		0.78
S15	Card collection		0.72
*P4*	*Understanding (iCHF)*	5.1	
S16	Only relevant for chronic diseases		0.80
S17	Health is in the hands of God		0.74
S18	iCHF is for government workers		0.39
*P5*	*Recommendation (iCHF)*	5.1	
S19	iCHF representatives		0.85
S20	Relatives and friends		0.83
*P6*	*Knowledge (iCHF)*	4.8	
S21	Awareness about the iCHF premium		0.78
S22	The iCHF benefits are clear to me		0.51
S23	Knowledge about the iCHF scheme		−0.39
S24	The iCHF Premium is affordable		0.38
*P7*	*Awareness (iCHF)*	4.7	
S25	iCHF is for irregular incomes earners		−0.65
S26	I know people that are members of iCHF		0.57
S27	Current needs are prioritized		0.44
S28	iCHF is like paying taxes		0.42

Source: Authors’ calculation of PCA based on primary data

Note: Primary data were collected from two rural districts of Dodoma region (Bahi and Chamwino)

The findings for the factor analysis (*FA)* are presented in Table 5. For this method, four factors were identified that accounted for 91% of the explained variation. All four factors fulfilled the factor-loading criteria and in sum, the 4 factors include 22 of the 33 statements. The number of statements belonging to each factor varied from two to eight. The most significant changes, compared with *PCA*, are that Preferences (*P2*) and Understanding (*P*4) now are collapsed into one single factor denoted as Preferences/Understanding (*F2*). Furthermore, we observe that; (i) an additional provider quality dimension (facilities, S29) becomes part of Quality (*F1*), (ii) the affordability statements (S*11* and S*24*) are now ignored, and, (iii) two of the three factors that measured the degree of understanding (Knowledge and Awareness) are now excluded.

**Table 5:** Factor Analysis (*FA*): Household Perceptions towards iCHF

S/N	Factors and included statements	The explained variance (%)	Factor Loadings
*F1*	*Quality (health care services)*	34.1	
S1	Healthcare services		0.74
S2	Healthcare personnel		0.71
S3	Long waiting time		−0.63
S4	Reasonable treatment time		0.60
S5	Discrimination of members		−0.55
S6	Availability of drugs		0.67
S29	Facilities (equipment)		0.33
*F2*	*Preferences/Understanding*	27.4	
S7	iCHF is a loss of money		0.50
S8	I save money in case of illness		0.50
S9	I borrow money in case of illness		0.60
s10	Prefer traditional healers		0.60
S12	Insurance brings bad luck		0.55
S16	Only relevant for chronic diseases		0.33
S17	Health is in the hands of God		0.43
S18	iCHF is for government workers		0.54
*F3*	*Convenience (iCHF accessibility)*	19.6	
S13	Office hours		0.69
S14	Opening location		0.66
S15	Card collection		0.53
S21	Awareness about the iCHF premium		0.31
S30	iCHF is a prepayment scheme		0.36
*F4*	*Recommendation (iCHF)*	9.9	
S19	iCHF representatives		0.59
S20	Relatives & friends		0.59

Source: Authors’ calculation of FA based on primary data

Note: Primary data were collected from two rural districts of Dodoma region (Bahi and Chamwino)

The three most important factors for *FA* are Quality (*F1),* Preferences/Understanding (*F2*), and Convenience (*F3)*, and they account for about 34, 27%, and about 20%, respectively, of the total variance. This means that the four most important factors identified for *PCA* (*P1-P4*) are also the most important ones for *FA*, however, for the latter two of the four factors are integrated into one single factor (Preferences/Understanding).

The various perception factors, together with household characteristics, are introduced as independent variables in multivariate regressions where iCHF membership status is the dependent variable. Based upon the statements belonging to each of the factors, we expect positive associations between membership and Quality (*P1* and *F1*), Convenience (*P3 and F3*) Knowledge (*P6*), and Recommendation (*P5* and *F4*) while we expect negative associations for Preferences (*P2*), Understanding (*P4*) and Preferences/Understanding (*F2*). As concerning the household characteristics, education, income, and household size are expected to increase the probability of being enrolled in the iCHF.

### Regression analysis

The logistic regression results are presented in Table 6. A total of fifteen variables influencing the household membership status were included in the first model and 12 variables in the second model. The first model included seven perception factors identified from PCA combined with eight household characteristics while the second model had 4 perception factors identified by FA and 8 household variables. From Table 6 we observe that 6 out of the 7 perception factors given *PCA* were significant (Awareness was non-significant) and 2 out of 8 household characteristics variables were significant. For *FA*, all 4 perception factors were significant and 2 of the household variables were significant.

**Table 6:** Multivariate Logistic Regression results of perception factors and household characteristics on membership status

Variables	Model 1: PCA Results	Model 2: FA Results
	OR* (SE)	OR* (SE)
Quality P1, F1	1.279 *** (0.101)	1.464*** (0.129)
Preferences P2, F2	0.614*** (0.052)	0.577*** (0.063)
Convenience P3, F3	1.402*** (0.128)	1.497*** (0.171)
Understanding P4	0.830 ** (0.061)	
Recommendation P5, F4	0.826*** (0.052)	0.843** (0.068)
Knowledge P6	1.390 *** (0.109)	
Awareness P7	1.075 (0.078)	
Household characteristics		
*Sex*		
Female	1	1
Male	0.753 (0.146)	0.753 (0.145)
*Age (years)*		
60+	1	1
40–59	0.571** (0.156)	0.567** (0.154)
26–39	0.459*** (0.136)	0.466*** (0.136)
18–25	0.582 (0.268)	0.562 (0.252)
*Education*		
Secondary and higher education	1	1
Primary education	1.029 (0.325)	0.918 (0.282)
No education	1.268 (0.489)	1.049 (0.394)
*Marital status*		
Unmarried	1	1
Married	1.165 (0.257)	1.193 (0.263)
*Family size*		
≥ 10	1	1
7–9	0.760 (0.361)	0.751 (0.357)
4–6	0.736 (0.338)	0.737 (0.336)
≤ 3	0.677 (0.325)	0.679 (0.327)
*Religion*		
Muslim	1	1
Christian	1.119 (0.289)	1.162 (0.296)
*Occupation*		
Non-farmers	1	1
Farmers	0.951 (0.202)	0.968 (0.206)
*Income (in TZS)*		
1.000.000 and higher	1	1
500.000–999.999	0.683 (0.562)	0.599 (0.488)
100.000–499.999	0.480 (0.349)	0.416 (0.299)
50.000–99.999	0.357 (0.264)	0.317 (0.231)
0–49.999	0.267* (0.198)	0.218** (0.159)
Number of observations	722	722
Log-likelihood	− 391.5037	− 396.7734
Likelihood ratio test	84.02	77.42
Prob >chi2	0.000	0.000
Pseudo R2	0.1145	0.1028

Source: Authors’ calculation of logistic regression based on primary data

Notes: (1) Primary data were collected from two rural districts of Dodoma region (Bahi and Chamwino) (2) Significance level: ***(*p* ≤ 0.01); **(*p* ≤ 0.05); *(*p* ≤ 0.1)

The signs of the factors are as expected except for Recommendation (*P5* and *F4*). The factors that appear to be most important, evaluated by significance levels and the size of the odds-ratios, are Preferences, Convenience, Knowledge, and Quality for *PCA* while for *FA* they are Convenience, Preferences/Understanding, and Quality.

Three factors for PCA and two factors for FA have a positive association with enrolment status. For PCA, the odds of a household being enrolled into iCHF, increase by 28, 40, and 39% as Quality, Convenience, and Knowledge, respectively, become higher. For FA, the odds of enrolling in the iCHF scheme increase by 46% (Quality) and 49% (Convenience). Factors that are decreasing the odds of enrolling (both for PCA and FA) are; Preferences, Understanding, and Recommendation.

We also observed that two of the eight variables (age and income) are statistically significant in both model 1 and model 2. The odds of being an iCHF member are 51, 58, and 44% lower for households whose respondent was aged between 18 and 25 years, 26–39 years, and 40–49 years relatively to households whose respondent is aged 60 years or older. Regarding household’s income, the odds of being insured by iCHF are 76% lower for households with income between 0 and 49,999 Tshs, relatively to households with income of 1,000,000 TZS or higher. Contrary to our expectations, household size and education level turned out insignificant.

## Discussion

We have applied principal component analysis and factor analysis methods to analyze the perception of households towards a community-based insurance scheme (iCHF). Both methods reduce many variables (statements) into fewer and more manageable variables or factors. *PCA* assumes there is no unique variance thus the total variance is equal to the common variance while *FA* assumes that the total variance can be partitioned into common and unique variances.

The results for the two methods differ somewhat for the number of factors identified and how much each factor explains the total variance. However, the most important perception factors are the same across the two methods; These were; Convenience (as exemplified by location and opening hours of iCHF offices), Quality (healthcare services), Preferences (the importance of alternative risk-reducing strategies such as saving and borrowing) and Knowledge.

Our findings partly contrast earlier studies on community-based insurance and household perception factors. Jehu-Appiah et al., (2012), in a study from Ghana, identified scheme factors (premiums, scheme benefits, and scheme convenience) as the most important perception factors [17]. In our study, the same factors, except for scheme convenience, were not important. Kansra and Gill (2017), in a study conducted in India, identified “lack of awareness and information about the insurance scheme” and “low and irregular income” as the most important perception factors [18]. In our study, however, the statements concerned with affordability (price-income statements) did not turn out as important. A possible explanation for this could be due to differences in study settings of the three studies. The study in Ghana was conducted in both rural and urban areas and the study in India was conducted in urban areas while this study was conducted in rural areas. As a result of differences in settings, the urban population might have different perceptions towards provider’s factors as compared to the rural population. This is because healthcare services in urban areas typically are of better quality hence being perceived more positive. This may explain why there were no statistical differences in the provider’s factors in the two studies and why the provider’s factors were the most significant ones in our study. Majority of the rural population have negative perceptions towards provider’s factors implying that if such factors are improved, more rural people will join the insurance scheme.

Using logistic regression analysis, we found that the quality of care, access to the iCHF offices, and preferences had the most significant influence on iCHF membership status. Furthermore, the presence or non-presence of household characteristics did not impact our results in important ways. The only socio-demographic variables that turned out significant, in combination with the perception factors, were age and income. However, the age groups 18–25, 26–39, and 40–49 years (economically active group) had lower odds of enrolling in the iCHF, relative to the aged 60 years or older. A possible explanation for this could be due to the positive association between age and healthcare utilization. Demand for healthcare services tends to increase with age. Surprisingly, education was not statistically significant for any of the regressions performed. Possible explanations for this finding are because; first, the scheme targets the informal sector most of whom are not highly educated. Secondly, when people increase their education level, they are more likely to be employed either by the Government or private sector that have different types of insurance (NHIF and PHI). As a result, those with primary education or no education are the ones who purchase the premium for iCHF. Also, the education level of the respondent was not representative of the education level of the household (the average education level). Furthermore, for the regression that considers household characteristics alone, gender was significant (*p* = 0.03), however, when including the perception factors, gender became insignificant. This last finding may suggest confounding effects between the perception factors and gender.

Our findings concerning provider quality indicate that people are more willing to purchase insurance if the quality of health care services is improved. This finding is consistent with results from other research conducted in Tanzania. Several studies have identified a positive association between quality of care and the enrollment into the predecessor of the iCHF scheme [10, 15, 29]. Similar findings have also been reported in Uganda [30] and Kenya [31].

Another interesting finding is that the statements about the role of prices (premiums) and low income (affordability) were not important predictors of enrollment. This suggests that purchasing power is not an important barrier for enrolling in the iCHF in Tanzania. The answer to one of the statements, not included in our factor analysis, seems to confirm this. From the survey it follows that 93% of the respondents strongly agreed or agreed to the following statement; “the ICHF scheme will become more important to me if additional health care expenditures were covered despite a corresponding increase in the premium.” Furthermore, 2/3 of all respondents agreed or strongly agreed with the statement “the iCHF premiums are affordable to me.”

Access to the iCHF offices (location, opening hours, and modality of collecting membership card) is the most important scheme factor in our analysis. This finding is in line with Winani (2015) who found that a longer distance between the community and the nearest CHF office acted as a barrier to enroll in the health insurance scheme in Tanzania [32]. Other studies from Africa also confirm such effects [17, 33]. The factor concerned with beliefs and alternatives, confirms as expected that, respondents that consider alternatives to insurance (saving and borrowing) and cure (traditional healers, health is in the hands of God) are less likely to be members of iCHF. The sign of the factor that includes recommendations from relatives, friends, and iCHF representatives turned out opposite of what was expected. A possible explanation is that the recommendations given to the respondents from family and friends are not very plausible, in this way affecting their enrolment decision negatively.

The results from the multivariate regressions performed by Jehu-Appiah et al. (2012) and Kansra and Gill (2017) confirm that the most important perception factors also became the most important determinants in the regression analyses [17, 18]. The study from Ghana found the benefits of the insurance scheme, the premiums, and convenience to be important while factors related to the quality of care were not associated with insurance scheme enrolment [17]. The study from India, on the other hand, identified a lack of awareness and low and irregular income as the most important determinants [18]. Thus, our findings differ from both studies since provider quality is important while affordability (income and premiums) is not important. As concerning household characteristics, our study identifies age and income to have some relevance, while in [17] most household characteristics (education, income, gender, age, and religion) became significant while [18] did not identify any household characteristics (gender, age, income, marital status, and education) as being significant. The two studies differ somewhat from our study since [17] surveys a mix of urban and rural populations with more than 60% of the respondents being males, while [18] surveys urban populations with 91% of the respondents being males. Our study, in contrast, study rural populations (mainly farming households) and 58% of the respondents were females.

From the similarities and contradictions of these findings, relative to the health financing policy implications, we learn that the scheme coverage for Tanzania is still low, more efforts to advertise/promote the scheme is needed. Moreover, the health system should also be improved as a means to increase the enrolment rate so that more people are protected. Furthermore, we learn that each country/society has different factors that drive people to enroll or not to enroll. As seen from the three countries, findings suggest that, in Tanzania, improvement in the quality of care (providers’ factors) is needed to influence enrolment decisions, from Ghana, scheme factors such as convenience, benefit package, and affordability are the most important factors to influence enrolment decision. In India, Information, knowledge, and income are important factors to influence decisions.

### Limitations and strengths

A cross-sectional study is not without some limitations. This study was conducted in two districts of Tanzania within one region, which makes it difficult to generalize the interpretation of the results to the other regions implementing the iCHF scheme. We, therefore, argue that the findings should be interpreted with some caution. Furthermore, a majority of the respondents were female (58%) thus introducing the possibility of gender bias. We can not rule out that female respondents differ from male respondents along some dimensions. However, our survey had a participation rate equal to 100%, meaning that we are not confronted with any selection bias.

## Conclusions and recommendations

Our study shows that household perceptions influence households’ decision to enroll in CBHIs. It was interesting to note that provider-related factors such as the quality of health care play an important role while affordability (income and premiums) does not seem to play a significant role. These findings suggest that efforts to achieve a higher enrolment rate in Tanzania should focus on improving the quality of healthcare services in terms of drug availability, reduced waiting time, and better services.

Poor perceived quality of care emerged as a significant barrier for household decision to enroll in iCHF. Majority of respondents had poor perceptions of quality of care and they were not satisfied with services received at the health facility. Several measures must be put in place to improve the quality of care by hiring more healthcare providers and by increasing the number of medical supplies used at the facilities.

The improvement of the quality of health services alone might not guarantee an increase in the enrolment rate in the iCHF. This study identified beliefs in traditional healers and other life preferences such as saving for the future to be the other important factors that deter people from buying health insurance premiums. Therefore, raising awareness to the community on the importance of having health insurance is still of paramount importance.

Furthermore, the unimportant role of affordability suggests that, for most households, income and premiums are less likely to be the barriers to enrolment into the community-based insurance scheme (iCHF). This in turn implies that the premium might be raised with less worry of experiencing a significant decline in the enrolment rate and the corresponding increase in revenues can be invested into improving the quality of services as well as extending insurance coverage. In this way, policymakers will ensure that community expectations concerning the iCHF scheme are met, thus increasing the future enrolment rate. However, despite the insignificance of affordability factors (premiums and income) for the whole study group, policymakers should also pay attention to the groups being most vulnerable to out-of-pocket health care expenditures. For this group, premium subsidization and more flexible payment arrangements should be considered.

## Supplementary Information

**Additional file 1.** English language version of the questionnaire guide used in this study.

## Data Availability

The datasets used and/or analyzed during the current study are available from the corresponding author on reasonable request.

## Abbreviations

CHF: Community Health Fund
iCHF: Improved Community Health Fund
CBHIs: Community –Based Health Insurance Schemes
LMICs: Low and Middle-Income Countries
NHIF: National Health Insurance Fund
PHI: Private Health Insurance
PCA: Principal Component Analysis
FA: Factor Analysis
IMIS: Insurance Management Information System
Tshs: Tanzanian shillings
USD: United State Dollar
WHO: World Health Organization
KMO: Kaiser Meyer-Olkin measure
SD: Standard deviations
SDGs: Sustainable Development Goals

## Footnotes

1 Study [18] applies factor analysis (FA), while study [17] applies principal component analysis (PCA).

2 A household is defined as one or more people, related or unrelated, who share meals and who live in the same dwelling unit [19, 20]. For this study, the household definition had to be modified since, in some cases, household members from more than one household come together to join an iCHF household.

3 Both Understanding, Knowledge and Awareness are dominated by statements concerned with measuring the respondents’ understanding of the iCHF scheme, and to what degree they are informed about the contract terms.

## References

[1] WHO, The World Bank IB for R and D. Tracking Universal Health Coverage: 2017 Global Monitoring Report. Geneva; 2017.

[2] WHO. Global Spending on Health: A World in Transition 2019. Global Report. Geneva; 2019.

[3] Xu K, Soucat A, Kutzin J, Brindley C. Public Spending on Health : A Closer Look at Global Trends. Geneva: WHO; 2018.

[4] WHO (2019). Questions and Answers on Universal Health Coverage.

[5] Basaza R, Criel B, Van der Stuyft P (2007). Low enrolment in Ugandan community health insurance schemes: underlying causes and policy implications. BMC Health Serv Res.

[6] Devadasan N, Ranson K, Van Damme W, Acharya A, Criel B (2006). The landscape of community health insurance in India: an overview based on 10 case studies. Health Policy.

[7] Dror DM, Jacquier C (1999). Micro-insurance: extending health insurance to the excluded. Int Soc Secur Rev.

[8] Kalolo A, Radermacher R, Stoermer M, Meshack M, De Allegri M (2015). Factors affecting adoption, implementation fidelity, and sustainability of the redesigned community health Fund in Tanzania: a mixed methods protocol for process evaluation in the Dodoma region. Glob Health Action.

[9] Chee G, Smith K, Kapinga A (2002). Assessment of the community health find in Hanang District, Tanzania.

[10] Waheke W. Effects and Challenges of Community Health Fund on Accessibility to Health Care Services: A case of Songea District (Doctoral dissertation, Mzumbe University.) Tanzania. 2015.

[11] Aggarwal A (2011). Achieving equity in health through community-based health insurance: India's experience with a large CBHI programme. J Dev Stud.

[12] Kuwawenaruwa A, Borgh J. Health insurance schemes in Tanzania. Dar es Salaam: Ifakara Health Institute; 2012. (11).

[13] NBS (2013). Population Distribution by Age and Sex.

[14] Anaeli A. Access to health care in Tanzania: An exploration of Community Health Fund enrolment decision process and patient satisfaction by insurance status. Denmark: PhD Dissertation: Aarhus University; 2013.

[15] Kamuzora P, Gilson L (2007). Factors influencing implementation of the community health Fund in Tanzania. Health Policy Plan.

[16] MoHCDGEC. Summary of the Budget Speech for the Financial Year 2018/19 Delivered by the Minister of Health, Community Development, Gender, Elderly and Children. Tanzania Ministry of health. Dar Es Salaam; 2018.

[17] Jehu-Appiah C, Aryeetey G, Agyepong I, Spaan E, Baltussen R (2012). Household perceptions and their implications for enrolment in the National Health Insurance Scheme in Ghana. Health Policy Plan.

[18] Kansra P, Gill HS (2017). Role of perceptions in health insurance buying behaviour of workers employed in informal sector of India. Glob Bus Rev.

[19] Smeeding TM, Weinberg DH (2001). Toward a uniform definition of household income. Rev Income Wealth.

[20] Sullivan AO, Sheffrin SM, Perez SJ, Onor C, Aura M, Eera M (2014). Survey of Economics Principles, Applications, and Tools.

[21] Dror DM, Shahed Hossain SA, Majumdar A, Koehlmoos TLP, John D, Panda PK (2016). What factors affect voluntary uptake of community-based health insurance schemes in low- and middle-income countries? A systematic review and meta-analysis. PLoS One.

[22] Macha J, Kuwawenaruwa A, Makawia S, Mtei G, Borghi J (2014). Determinants of community health fund membership in Tanzania: a mixed methods analysis. BMC Health Serv Res.

[23] Minyihun A, Gebregziabher MG, Gelaw YA (2019). Willingness to pay for community-based health insurance and associated factors among rural households of Bugna District, Northeast Ethiopia. BMC Res Notes.

[24] Kuwawenaruwa A, Macha J, Borghi J (2011). Willingness to pay for voluntary health insurance in Tanzania. East Afr Med J.

[25] Costello AB, Osborne J (2005). Best practices in exploratory factor analysis: Four recommendations for getting the most from your analysis. Pract Assess Res Eval.

[26] Antony GM, Rao KV (2007). A composite index to explain variations in poverty, health, nutritional status and standard of living: use of multivariate statistical methods. Public Health.

[27] Williams R, Dame N (2015). Measurement Error 2 : Scale Construction.

[28] Kaiser HF (1960). The application of electronic computers to factor analysis. Educ Psychol Meas.

[29] Macha RR (2015). Community based health insurance schemes and protection of the rural poor: empirical evidence from Tanzania. Afr J Econ Rev.

[30] Basaza RK, Criel B, Van der Stuyft P (2010). Community health insurance amidst abolition of user fees in Uganda: the view from policy makers and health service managers. BMC Health Serv Res.

[31] Barasa EW, Mwaura N, Rogo K, Andrawes L (2017). Extending voluntary health insurance to the informal sector: experiences and expectations of the informal sector in Kenya. Wellcome Open Res.

[32] Winani S (2015). Community health fund scheme in Tanzania: exploration of its challenges and opportunities in contribution towards Universal health coverage. J Appl Microbiol.

[33] Carrin G, Waelkens MP, Criel B (2005). Community-based health insurance in developing countries: a study of its contribution to the performance of health financing systems. Tropical Med Int Health.

