# Supplementary material for: Do household perceptions influence enrolment decisions into community-based health insurance schemes in Tanzania?
Source: BMC Health Serv Res. 2021 Feb 19;21:162. doi: 10.1186/s12913-021-06167-z (PMC7893739; doi:10.1186/s12913-021-06167-z)
Supplement: Supplementary file 1 — Additional file 1. English language version of the questionnaire guide used in this study. [file 12913_2021_6167_MOESM1_ESM.doc]

**Questionnaire guide for the study titled:** *Does Household Perceptions Influence Enrolment Decisions into Community-Based Health Insurance Schemes in Tanzania?*

**Section A: Socio-Demographic and Economic Information of the Respondents**

| 1. Age (in years) | 2. Sex  Male…...…1  Female.….2 | 3. Marital status  Single …………...1  Married.................2  Living together….3  Divorced...............4  Widowed..............5  Separated………..6 | 4. How many people, including yourself, live in this household?  …………….. | 5. How many members of your household are less than 14 years of age?  ……………. |
| --- | --- | --- | --- | --- |
| 6. Your highest level of education being completed  No formal education………...…….1  Primary school ……………………2  Ordinary level Secondary school …3  Advance level secondary school.….4  Vocational training………………...5  College ………................................6  University……………………..…..7 | | 7. Your religion  Christian............1  Muslim..............2  Traditional...…..3  None believer…4  Other (specify)..5 | 8. Your Main Occupation?  Farmer……………1  Livestock breeder...2  Retired …………...3 Craftsman……...….4 Merchant………….5  Civil Servant……...6  Daily wage laborer..7  Not earning any income…………….8  Other (specify)…....9 | 9. What is the average monthly income of your household?  (*This should be the sum of what all household members bring in taken together)*  …………...Tshs |

Section B: Membership Status

| 10.  Are you enrolled into the iCHF scheme?  Yes……….1  No………..2 (go to 14) | 11.  How many household members are registered members of the iCHF scheme?  .............number | 12.  For how long time have you/your household been members of the iCHF scheme?  …….Months/Years | 13.  Are you/your household planning to stay enrolled into the iCHF scheme in the future?  Yes……1  No…….2 |
| --- | --- | --- | --- |
| 14. Non-members  Have you/your household been former members of the CHF/iCHF scheme?  Yes………1(go to 16)  No……….2 | 15. Non-members  Do you/your household consider to become a future member of the iCHF scheme?  Yes….1  No.….2 | 16.  Are there any household members being members of any other health insurance scheme than the iCHF?  Yes……1  No…….2 (go to sec C) | 17.  What type of insurance scheme?  NHIF………………………...1  PHI…………………...…...…2  SHIB…………………...…....3 |

**Section C: Perception Towards the iCHF Scheme, its’ Management and the Role of Healthcare Providers**

| **In the following, you will be confronted with various statements. For each statement you will be asked to express to what degree you agree. This is done by reporting a number between 1 to 5 where 1 means that you strongly disagree while 5 means that you strongly agree** | | **1**  **Strongly Disagree** | **2**  **Disagree** | **3**  **Neutral** | **4**  **Agree** | **5**  **Strongly agree** |
| --- | --- | --- | --- | --- | --- | --- |
|  | | | | | | |
| S1 | The health care providers supply services of an adequate quality to the iCHF members |  |  |  |  |  |
| S2 | The quality of health care personell is satisfactory |  |  |  |  |  |
| S3 | The waiting time for healthcare services is too long for iCHF members. |  |  |  |  |  |
| S4 | Members of the iCHF are receiving treatment within a reasonable time horizon |  |  |  |  |  |
| S5 | At health care facilities, iCHF scheme members are being discriminated against |  |  |  |  |  |
| S6 | Being an iCHF member improves drug availability |  |  |  |  |  |
| S7 | Health insurance is basically a waste of money |  |  |  |  |  |
| S8 | I prefer to save money rather than to spend them on health insurance |  |  |  |  |  |
| S9 | I prefer to stay uninsured and borrow money to cover health expenditures |  |  |  |  |  |
| S10 | I prefer to visit tradition healers (traditional medicine rather) rather than enrolling into an insurance scheme |  |  |  |  |  |
| S11 | The benefits from iCHF is too low relatively to the premium charged |  |  |  |  |  |
| S12 | The purchase of health insurances may bring bad luck to the purchasers |  |  |  |  |  |
| S13 | The opening hours of the iCHF scheme district office are convenient |  |  |  |  |  |
| S14 | The location of the iCHF scheme district office is convenient |  |  |  |  |  |
| S15 | The collection of the iCHF insurance cards is convenient |  |  |  |  |  |
| S16 | Only households with family members with chronic diseases should enroll into a health insurance scheme |  |  |  |  |  |
| S17 | Health is a matter of fate (in the hands of God), thus health insurance schemes cannot help me deal with the consequences of bad health |  |  |  |  |  |
| S18 | The iCHF scheme is only relevant for those employed in the public sector |  |  |  |  |  |
| S19 | I have never been approached by iCHF representatives wanting me to become a member of the iCHF scheme |  |  |  |  |  |
| S20 | My decision to enroll, or not enroll, into the iCHF scheme depends heavily on the advices given by friends, relatives and colleagues. |  |  |  |  |  |
| S21 | I am fully aware of both the premium size and the registration fee of the iCHF scheme |  |  |  |  |  |
| S22 | The benefits from the iCHF scheme are clear to me |  |  |  |  |  |
| S23 | I do not have much knowledge about the iCHF scheme |  |  |  |  |  |
| S24 | The iCHF premiums are affordable to me |  |  |  |  |  |
| S25 | Health insurance schemes are particularly suitable for people with irregular incomes |  |  |  |  |  |
| S26 | Many people that I know well are members of the iCHF scheme |  |  |  |  |  |
| S27 | Generally, I tend to give importance to current needs over future problems |  |  |  |  |  |
| S28 | Paying premiums to an health insurance scheme is no different from paying taxes to the government |  |  |  |  |  |
| S29 | The health care facilities in my district have adequate equipments and rooms |  |  |  |  |  |
| S30 | Health insurance schemes are prepayments for health care services |  |  |  |  |  |
| S31 | The iCHF scheme managers are generally trustworthy |  |  |  |  |  |
| S32 | In order to increase the iCHF enrollment rate, the attitude of the health care personnel needs to be improved |  |  |  |  |  |
| S33 | People that I know well, such as relatives, friends and colleagues, have given me the advice to enroll into the iCHF scheme. |  |  |  |  |  |
| S34 | The iCHF scheme will become more important to me if additional health care expenditures were covered by the scheme despite a corresponding increase in the premium |  |  |  |  |  |
| S35 | The iCHF scheme is especially valuable if all household members are covered by the scheme |  |  |  |  |  |
| S36 | In order to enroll into the iCHF scheme you need a sufficient amount of cash |  |  |  |  |  |
| S37 | Low income makes it hard to give priority to the purchase of health insurance |  |  |  |  |  |
| S38 | Community members who cannot afford insurance premiums should be enrolled into the scheme without having to pay any premiums |  |  |  |  |  |
